# A case report of cognitive behavioral therapy for social anxiety

**DOI:** 10.3389/fpsyg.2025.1520581

**Published:** 2025-05-27

**Authors:** Feifei Xu, Hang Zhang

**Affiliations:** ^1^School of Humanities and International Education Exchange, Anhui University of Chinese Medicine, HeFei, China; ^2^School of Psychology, Zhejiang Normal University, Jinhua, China

**Keywords:** cognitive behavioral therapy, social anxiety disorder, anxiety-related disorders, self-rating anxiety scale, exposure therapy

## Abstract

**Objective:**

To develop a case conceptualization consultation plan for social anxiety cognitive behavioral therapy (CBT).

**Methods:**

Firstly, according to the process and requirements of case conceptualization, the content of the case is presented to the customer in the form of conceptual model diagram. Subsequently, according to the specific problems raised in each part, targeted interventions were implemented by means of exposure, cognitive reconstruction, inhibition of safe behaviors, etc., and the intervention was divided into eight stages, all the patients were assessed before and after the consultation.

**Results:**

Before the intervention, the patient SCL-90 scale, total score 190, somatization: 2.1, interpersonal sensitivity: 2.2, depression: 2.1, and anxiety: 2.7. Fear Factor Score: 2.4, other factor score <2, positive item 45, significant anxiety symptoms; SAS: score 65; social avoidance and distress scale-SAD-SAD total score of 20; It showed that the patients had significant social anxiety in social interaction. After the intervention, Scl-90: Total score of 140, score of <2 for each factor, number of positive items of 30, and all indicators returned to normal: SAS: standard score of 49: SDS: Standard score of 38, no depressive mood. The total score of SAD was 14, with a score of 5 for avoidance and 9 for anxiety, indicating improvement in social anxiety.

**Conclusion:**

The case conceptualization approach in social anxiety CBT consultations facilitates patients with social anxiety issues in understanding their problems, demonstrating practical application value in psychological counseling.

## 1 Introduction

Social Anxiety Disorder (SAD), also known as social phobia, has a lifetime prevalence ranging from 3 to 14%, significantly impairing the social functioning of patients (Sareen and Stein, 2000). According to the latest psychological survey conducted by the World Health Organization in 2017, the lifetime prevalence of SAD reached 4% (Stein et al., 2017). Adolescence is a particularly vulnerable period for the development of social anxiety (Rosellini et al., 2013). When adolescents suffer from SAD, it not only jeopardizes their mental health and disrupts their interpersonal relationships but also leads to adverse consequences such as declining academic performance and reduced quality of life (Brook and Willoughby, 2015). Adolescents with SAD often avoid contact with others due to fear of negative outcomes, exhibiting frequent avoidance behaviors and lacking social skills in interactions with strangers. These behaviors, in turn, exacerbate feelings of self-pity or inferiority, fostering a reduced sense of meaning in life (Creed and Funder, 1998). Furthermore, the Social Cognitive Development Model suggests that as individuals progress through childhood and adolescence, with the growth of self-awareness, there is a decrease in fear of physical harm and an increase in fear of social evaluation. Consequently, individuals become increasingly concerned with how others perceive and evaluate them in social interactions, predisposing them to the fear of negative evaluations (Westenberg et al., 2004). People with social anxiety disorder are often shy when meeting new people, quiet in groups, and introverted in unfamiliar social situations. When they interact with others, they may or may not show obvious signs of discomfort (e.g., blushing, not making eye contact with others), but will always present with strong emotional or physical symptoms, and they may not show signs of discomfort when they interact with others, or both (e.g., fear, racing heart, sweating, tremors, difficulty concentrating). They crave the company of others but avoid social situations for fear of being found unlikable, stupid or boring. As a result, they avoid speaking in public, expressing their opinions and even avoiding close contact with their peers, which in some cases may lead to these people being wrongly labeled as snobs. People with social anxiety disorder are typically characterized by low self-esteem and high levels of self-criticism (Bj and Mb, 2004). The researchers found overactivation of fear circuits in SAD (amygdala, insula, anterior cingulate, and Prefrontal cortex). In addition, task-related functional studies revealed overactivation of medial parietal and occipital regions (posterior cingulate, precuneus, precuneus) in SAD, which may contribute to the development of SAD, and decreased connectivity between parietal and limbic and executive network regions (Brühl et al., 2014). These findings suggest that a more comprehensive approach to the rehabilitation of people with social anxiety may offer more powerful benefits. At the same time, the treatment of social anxiety disorder also needs more exploration.

Cognitive behavioral therapy (CBT) has demonstrated promising effects in treating anxiety (Reuman et al., 2021; Bilek et al., 2022) rooted in a conceptual model of individual anxiety experiences and their maintenance (Clark, 1999). CBT is a time-limited, moment-to-moment approach to psychotherapy that teaches patients the cognitive and capacity skills they need to function adaptively in the interpersonal and internal world. It is a joint effort by the therapist and the patient, who form a collaborative team to address the patient’s concerns. CBT also emphasizes empirical evidence of efficacy in controlled studies. The main cognitive behavioral therapy categories used to treat social anxiety disorder include exposure, cognitive restructuring, relaxation training, and social skills training (Carpenter et al., 2018). Results from a randomized clinical trial study of 91 adolescents with anxiety disorders who participated in CBT showed that adolescents achieved similar benefits both after treatment and during the 1-year follow-up period; however, adolescents with symptoms or diagnoses of social anxiety improved significantly less during the 7.4-year follow-up period (Kerns et al., 2013). Randomized controlled trials and meta-analyses have shown the efficacy and effectiveness of individual CBT for ARD (Mayo-Wilson et al., 2014). While many adults and adolescents can benefit from CBT, there is still a substantial proportion of individuals (ranging from 9 to 50%) who refuse CBT treatment, drop out early, do not respond to CBT, or are unable to maintain long-term effects (Taylor et al., 2012). Consequently, research on how to improve CBT for anxiety and related disorders represents an important research objective.

In psychological counseling and therapy, case conceptualization has increasingly been integrated, particularly in the context of CBT. This approach is applicable to CBT, despite the fact that specific symptoms of different anxiety disorders vary. While they diverge in specific manifestations, anxiety disorders share some common ground, namely their core symptoms. Firstly, individuals with anxiety disorders exhibit varying degrees of psychological symptoms. Secondly, all anxiety patients harbor irrational beliefs in their cognition. These commonalities lead us to the conclusion that anxiety and anxiety disorders are not synonymous, and they facilitate the possibility of conceptualizing anxiety cases through a unified model. The researchers found that negative biases, inaccuracies and rigid beliefs are thought to play a key role in anxiety disorders. In a sample of 47 SAD patients treated with CBT, investigators measured maladaptive interpersonal beliefs and the emotional and behavioral components of social anxiety at baseline and after treatment completion. Results showed that maladaptive interpersonal beliefs were associated with social anxiety at baseline and after treatment completion; treatment-related reductions in maladaptive interpersonal beliefs fully explained reductions in social anxiety after CBT. The evidence suggests more ways to treat SAD (Matthew Tyler et al., 2012). Boschen and Oei (2008) have proposed the Cognitive Behavioral Case Formulation Framework (CBCFF), which serves as such a comprehensive model comprising seven primary components: approach behaviors and triggers, hypervigilance, experiential information, neuroticism, reduced self-efficacy, anxiety-reducing behaviors and safety signals, and anxiety reduction. Through the formulation of CBCFF, this study helps clients understand and solve problems encountered, and presents the process of treatment through cases, with a view to helping future research to improve the efficiency of treatment, it also provides more support for the rehabilitation of people with social anxiety. This paper aims to utilize this model to develop individualized counseling plans for individuals with social anxiety disorder and comprehensively evaluate their effectiveness.

## 2 Methods

### 2.1 Case basic information

#### 2.1.1 Case basics

Patient A is a middle school second-year male student with no family history of mental illness, no history of major injuries, and no bad habits.

#### 2.1.2 Personal growth experience

Caller was born in ordinary families, working-class families, Cesarean section, normal development, living with their parents, has a brother, family financial situation is not good. Parents demanding, less emotional communication with children, visitors introverted character results better, in the local key secondary school, but learning interest is not great. Seldom takes the initiative to answer questions in class.

September 2022 has been experiencing significant discomfort since the middle school. He felt unable to adapt to the school life, especially the interpersonal relationship with his roommates, which caused him considerable trouble. In May, a 2023 failed in his dormitory hygiene test after he forgot to take out the garbage, leading to an argument with the dormitory director. During the argument, the dormitory director made the caller cry with excessive abuse, he felt ostracized by everyone around him. After the caller and the dormitory leader alone will feel nervous, flustered, chest tightness, palms sweating, unable to concentrate. Later, I became nervous around anyone in the dorm, and I was reluctant to participate in activities, fearing that the teacher would ask me questions in class, and that I would feel ridiculed and targeted by the teacher and other students.

In order to get a positive impression from their peers, visitors often seek approval in conversation, show a submissive attitude, and refrain from expressing their true selves for fear of being unappreciated. This inner conflict leads to a sense of frustration and helplessness as he grapples with the complexities of dorm life. These social anxieties manifest themselves physically in conversation, with symptoms such as nervousness, sweating, a racing heart and difficulty breathing, leading to headaches, difficulty concentrating and lower grades. In school and family life, the visitor would feel that everyone around him was paying attention to him. With the corresponding evaluation, the visitor reported feelings of anxiety, worry, and fear, so that he avoided going back to the dormitory, they often choose to go home and even consider changing classes or taking time off from school. This is also the case outside of school settings, such as in addition to communication with their parents is relatively smooth, the visit to communicate with friends and acquaintances expressed anxiety, fear of their performance is not good; They are difficult to enter the unfamiliar environment, it is difficult to maintain emotional stability. This kind of nervous uneasiness seriously affects the sleep and study of the caller. After verification with parents and classmates, it was found that the real situation was not consistent with the visitors’ statements, and the social-related threats did not reflect the real situation. The March 2024 realized the consequences and went to a local psychiatric hospital for a checkup. Doctors diagnosed him with social anxiety disorder, which is linked to anxiety and socializing, the Doctor suggested psychological treatment and came forward. The disclosure of information related to this study was done with the consent of the case and his parents, who signed informed consent and personal information disclosure forms, agreeing to publish the information.

#### 2.1.3 First impressions of consulting

At the first meeting, the consultant observed that patient a had a normal appearance, spoke slowly and low, frowned heavily, had stiff body language, rarely looked up when speaking, and avoided eye contact with the consultant. These non-verbal cues hint at underlying emotional distress.

#### 2.1.4 Function

*Mental state*: conscious, normal thinking, irritability, sometimes depressed mood, anxiety and tension, self-control but emotional instability, words and deeds consistent, stable personality.

*Physical state*: nearly 6 months of poor sleep, appetite is not strong.

*Social function*: apathy between classmates, avoid social intercourse, easy to be distracted in class, poor attendance.

#### 2.1.5 Diagnosis

Social anxiety disorder was diagnosed according to the Diagnostic and Statistical Manual of Mental disorders fifth Edition (DSM-5).

#### 2.1.6 Psychological test results

1. Scl-90: Total Score 190, somatization: 2.1, interpersonal sensitivity: 2.2, depression: 2.1, anxiety: 2.7. Terror Factor Score: 2.4, other factor scores were <2, positive items 45, anxiety symptoms were obvious;
2. SAS: Standard Score 65; indicates moderate anxiety.
3. SDS: Standard Score 61, showing mild depression.
4. The total score on the social avoidance and distress scale (SAD) was 20, with 9 on Avoid subscale and 11 on the distress subscale. It is worth noting that SAD scored 11 out of the normal range, further confirming that visitor a has significant social anxiety in interpersonal relationships, which is manifested as obvious anxiety and avoidance behavior.

#### 2.1.7 Consulting plan

With the informed consent of the patient, the psychologist and the patient collaboratively devised a consultation plan encompassing a total of eight sessions, conducted once a week for an hour each. In order to ensure the effectiveness of treatment and eliminate the therapist’s treatment bias, with the consent of the patient, the therapist communicates monthly with his or her supervisor to discuss the problems encountered and make program adjustments.

### 2.2 Consultation process

**Table 1:** 

	Time	Treatment content
Phase 1 (Session 1)	2024.03–2024.04	Initial Interview
Phase 2 (Session 2–3)	2024.04–2024.05	Case Conceptualization Process
Phase 3 (Session 4–7)	2024.05–2024.07	Intervention Techniques
Phase 4 (Session 8)	2024.07–2024.08	Consultation Closure

#### 2.2.1 Phase 1 (session 1): initial interview

- Gather general information from the patient and identify the presenting problem.
- Jointly establish the consultation plan and establish a preliminary therapeutic relationship.

In the first stage, the therapist tried to establish a relationship with the patient, and the patient had a strong motivation to seek help and good compliance.

#### 2.2.2 Phase 2 (sessions 2–3): case conceptualization process

Beginning with the second session, the psychologist introduces the case conceptualization of anxiety disorders proposed by Boschen and Oei (2008) to the patient. The therapist worked with the patient to complete the conceptual part of the case, the therapist helped the patient understand his problem behavior and tried to complete the part together, and the patient had good compliance.

- The therapist first presents the Cognitive Behavioral Case Formulation (CBCF) model for anxiety disorders.
- The therapist briefly introduces the key components of the model. After the patient comprehends the essential content of CBCEF, the main aspects of the case are conceptualized, ensuring the patient’s understanding.

This structured approach ensures that the consultation plan addresses the patient’s specific needs and concerns, fostering a collaborative and effective therapeutic process.

*Conceptualization*: the process of conceptualization involves the patient developing a unique understanding of their own problems. In other words, it enables the patient to form a distinct mental framework around their anxiety issues, providing clarity on their formation and progression. This insight is crucial for addressing the underlying causes and maintaining factors of the anxiety.

Patient A’s case conceptualization highlights the interrelationship between his interpersonal difficulties, reluctance to reside in the dormitory, and frequent homecoming. This avoidance behavior has heightened his sensitivity toward peers and the dormitory environment, fostering fears of isolation and rejection due to his perceived social ineptitude. This heightened sensitivity, coupled with fears of being ostracized, triggers headaches, impaired concentration, reduced learning efficiency, and, consequently, further anxiety.

The reinforcement cycle is evident: the inability to navigate interpersonal relationships strengthens Patient A’s conviction to adopt avoidance strategies (i.e., staying at home), which, albeit temporarily alleviating anxiety symptoms in a familiar and secure environment, ultimately perpetuates the problem upon his return to school.

**Figure 1:**
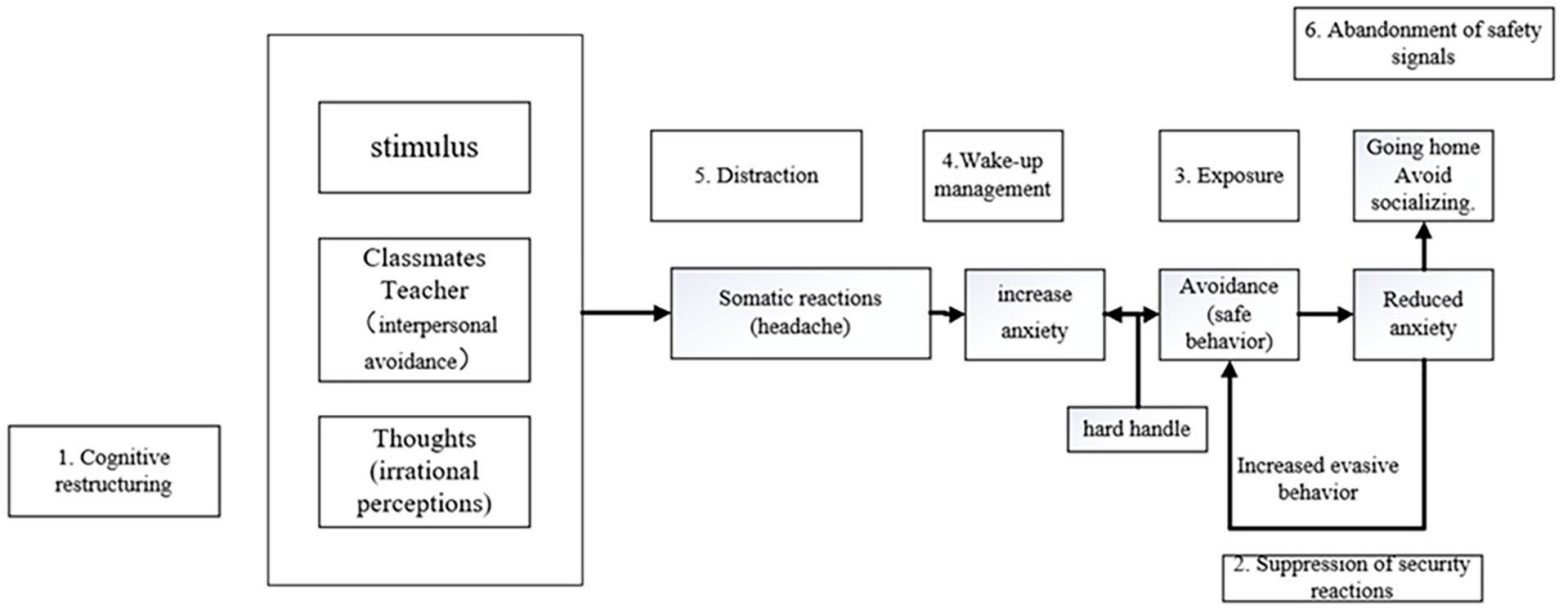
Case conceptualization process and corresponding counseling plan for patient A.

The importance of clear conceptualization: given that case conceptualization necessitates the acquisition of novel skills, it is imperative for Patient A to have a profound understanding of the process. The complexity of this endeavor necessitates multiple sessions, allowing for adjustments and refinements to the model as needed (Figure 1).

#### 2.2.3 Phase 3 (sessions 4–7): intervention techniques

After establishing the patient’s unique case conceptualization model, interventions are implemented based on the corresponding counseling methods for each component. The therapist instructs the patient to operate according to the relevant technical requirements, to perform exposure and cognitive reconstruction, during which the therapist focuses on the emotional changes of the patient, who has avoidance behavior during the exposure phase, but finally persisted, the compliance is good. Four primary intervention techniques were utilized in this case:

##### 2.2.3.1 Reminder and arousal management

Addressing the patient’s physical tension, accelerated heartbeat, and rapid breathing, the therapist facilitates the adoption of coping strategies. The therapist introduces progressive muscle relaxation and diaphragmatic breathing techniques, which the patient learns through daily practice. Through regular practice, the patient gradually learns to flexibly apply breathing relaxation techniques during heightened anxiety and to perform progressive muscle relaxation before sleep or during physical tension.

##### 2.2.3.2 Breathing relaxation as a means of attention shifting

Breathing relaxation, as a relaxation exercise, is also an effective method for diverting attention. It requires the patient to fully focus on their breathing, thereby shifting attention away from anxiety-provoking stimuli and reducing preoccupation with interpersonal situations.

##### 2.2.3.3 Exposure therapy

Exposure therapy serves as the cornerstone of behavioral techniques to address anxiety in this case. The therapist encourages the patient to engage in exposure exercises targeting the maladaptive behavior of social avoidance. A hierarchy of exposure levels and specific contents (e.g., chatting with classmates, being rejected by peers, confronting anger from peers) is jointly established and practiced. Gradual exposure and repeated exposure to anxiety-provoking situations help reduce the patient’s anxiety levels upon entering such situations. The emphasis is on establishing appropriate exposure levels and content to ensure the effectiveness of exposure in mitigating anxiety. Moderate punishment (e.g., placing a hand in cold water) is agreed upon for avoidance behaviors, coupled with rewards for exposure attempts. Exposure practice is incorporated into homework assignments to facilitate continuous progress and confidence-building.

##### 2.2.3.4 Cognitive restructuring

Cognitive-Behavioral Therapy (CBT) oriented therapists view anxiety as stemming from irrational beliefs, emphasizing the identification and modification of these beliefs as crucial.

The patient is first made aware that their own beliefs contribute to anxiety, fostering a sense of responsibility. Cognitive strategies such as challenging irrational beliefs, focusing on anxiety-provoking situations, and applying cognitive skills are employed to facilitate cognitive restructuring. Through these strategies, dysfunctional thinking patterns are transformed into more realistic, trustworthy, and positive ones. A “Thought Record” homework assignment is given to encourage the patient to document and dispute irrational beliefs encountered in daily life.

##### 2.2.3.5 Suppression of safety behaviors and abandonment of safety signals

Reducing safety behaviors and abandoning safety signals signify progress. In this case, the patient’s safety behavior involved avoiding anxiety-provoking situations (e.g., social interactions), while the safety signal was “going home.” Abandoning the safety signal does not imply ceasing all home visits but rather reducing them to normal frequencies unrelated to interpersonal issues. Enhanced self-efficacy and confidence in navigating anxiety-provoking situations are achieved through consistent exposure and successful coping.

#### 2.2.4 Phase 4 (session 8): consultation closure

The therapist and patient review the knowledge acquired and the patient’s adaptation progress. Patient compliance is good.

The consultant assesses the consultation’s effectiveness based on the consultation goals and the patient’s current status. The patient is informed that anxiety may persist or relapse post-consultation, necessitating psychological preparedness. Concrete measures for preventing relapse are discussed. The consultation concludes with a farewell between the therapist and patient.

## 3 Results

### 3.1 Evaluation of the effect of consultation

1. Self-evaluation of the patient: the anxiety and depression of the helper have been significantly improved, and the fear of avoidance in the dormitory has been transformed into relaxed communication with the dormitory director, in the class can also actively participate in classroom activities, can focus on learning and make friends, physical symptoms of anxiety in public and social settings (physical relaxation, breathing ease), mood (stable and self-regulating), and behavior (no more avoidance, and challenging yourself to do things you used to be afraid to do) improved after counseling. The lives of the callers in the dormitory are normal and classes are resumed.
2. Ratings from family members and classmates: the callers were less sensitive, held back, and occasionally made jokes.
3. Psychological assessment: SCL-90, SAS, SDS, SAD: Scl-90: Total score 140 points, each factor score is <2, the number of positive items 30, all indicators have returned to normal: SAS: Standard Score 49, standard score returned to normal: SDS: Standard Score 38, No Depression. The SAD total score was 14, with subscale scores of 5 for avoidance and 9 for anxiety.
4. The therapist’s assessment: the three-month follow-up showed that the social adaptability was significantly improved, the mental outlook was much better than that at the initial visit, the social anxiety was basically controlled, and the sleep state was good, the consultation basically achieved the expected goal.

The comprehensive evaluation shows that the psychological problems of the callers have been basically solved.

## 4 Discussion

This case study examines the CBCFF counseling approach for social anxiety cases. The results indicate that such a program can significantly mitigate the severity of anxiety issues from various angles. By integrating CBT with case conceptualization, some researchers contend that better counseling outcomes can be achieved, a notion echoed in the counseling plan of this case. Through case conceptualization, patients gain a deeper understanding of their issues, engage with the case conceptualization model diagram, and arrange corresponding counseling sessions accordingly. This plan combines the strengths of both case conceptualization and CBT orientation. Although the efficacy of case conceptualization in the field of psychological counseling and therapy is yet to be fully recognized, this case demonstrates that CBCFF may facilitate the recovery process of anxious patients in certain aspects.

In this case, the therapists developed a visual model diagram of their problem through the case conceptualization process. This diagram provides information on where problems originate, why problems lead to difficulties in their lives, how problems get worse and worse, and what sustains the vicious cycle. This map extends to the visitor’s early life experiences, beliefs, and thoughts, revealing that a well-crafted case conceptualization can shed light not only on the negative cycles of thought and behavior, but also on the ways in which a visitor’s life experiences, beliefs, and thoughts can influence his or her behavior, can also address key issues raised by visitors, for example: “Why does this keep happening to me?” “Why cannot I get rid of these problems?” “Can I change my situation?” and “What can I do to improve?” In this case, the CBCFF model translates a particular diagnosis into a corresponding intervention in each consultation session, highlighting its powerful Operability and practical applicability (Boschen and Oei, 2008).

Previous authors have keenly emphasized the supposed advantages of using Case formulation (CF)in psychotherapy. It is argued that the use of a systematic CF approach can provide clinicians with a theory-based framework to make inferences about the nature of visitors’ problems. A single CF allows for the provision of individual treatment plans instead of manual treatment delivery. The collaborative process of CF for cognitive behavioral therapy can also enhance the therapist and patient’s understanding of the current problem. Such personalized CF can also strengthen therapeutic alliances when collaboration is proposed. Furthermore, treatment outcomes could potentially be improved by proposing more specific and precise interventions (Bieling and Kuyken, 2003). This approach has also been found to be more useful than diagnosis-based treatment planning approaches (Jb, 1986) and can address concerns about the limitations of categorical diagnosis (Thomas and Cristina, 2018).

This case adopted the treatment strategy of “Cognitive reconstruction and exposure,” and achieved good therapeutic effect and therapeutic goal, which was consistent with the results of previous studies (Mayo-Wilson et al., 2014). The outcome of the treatment in this case has a lot to do with the successful implementation of the exposure, it is closely related to good treatment alliance, cognitive correction and psychological education in the early stage, and also inseparable from the treatment motivation of patients. In therapy, cognitive and behavioral techniques go hand in hand, complement each other and are equally important. In addition, instructing patients in therapy to focus on anxiety-provoking situations and to forgo safe behaviors would greatly increase the effect of exposure (Wells et al., 1995).

Anxiety generally decreases with increasing exposure, but some individuals experience considerable fluctuations in anxiety during a single exposure. Although anxiety during the first exposure was not significantly associated with outcome, the relationship between anxiety during exposure and outcome became stronger during subsequent exposures (Hayes et al., 2008). Observing the counseling process, it becomes evident that as exposure exercises yield results, patients become more adept at handling them. With reduced anxiety levels in interpersonal situations, punishment instances diminish. Exposure exercises allow patients to acquire accurate information through firsthand experience—social interactions are not as daunting as they had imagined, and spending time with classmates in dorms or classrooms is not as terrifying. Repeated exposures gradually accustom patients to the process of anxiety escalation, ultimately diminishing their anxiety levels. Successful coping during exposure strengthens patients’ self-confidence, revealing their capacity to navigate challenging situations, Reduce clinical symptoms, while increasing the associated positive cognition (Asbrand et al., 2020). They find themselves staying longer in interpersonal settings, engaging in fewer avoidance behaviors, and enhancing their sense of cognitive reappraisal self-efficacy (Goldin et al., 2012). Moreover, by improving poor cognition, individuals come to realize that not all situations are related to their mistakes and that not all people are hostile to them. Their thoughts tend to exaggerate reality, and when their irrational beliefs improve after therapy, they have the ability to think more rationally (Moloudi et al., 2022). The awareness of the difference between reality and self-perception can further help individuals to be more active in facing challenges, and virtual reality (VR) technology can be used as an alternative to individual image exposure. VR provides human-computer interaction, enabling patients to feel a sense of presence and immersion in a virtual environment, providing clinically anxious individuals with the opportunity to expose themselves to real-life scenarios, enabling them to learn more about the virtual world, and reduce their reactivity to anxiety-provoking cues. The impact of VR technology has been discussed in many studies and meta-analyses (Powers et al., 2013; Carl et al., 2019), and future studies could consider the combined use of this technology (Stefaniak et al., 2022). In essence, this is about replacing irrational beliefs with more rational and effective ones, thereby reducing anxiety, generating more positive thoughts, and further increasing self-efficacy in solving your own problems.

Given CBCFF’s emphasis on individuality, it is crucial to delve deeply into patients’ problems and the initial triggers of their symptoms, subsequently connecting various aspects in a comprehensive and systematic manner. For instance, while the patient initially mentioned issues with a specific roommate, the therapist, through exploration and expansion, recognized that the relationship problem extended beyond a single individual. A broader perspective enables targeted interventions and a more holistic understanding of the case. Consultants should be mindful that no case conceptualization model is flawless; continuously updating the model with new information is vital. Furthermore, the personalized and complex nature of case conceptualization necessitates rich practical experience on the part of the consultant, who should also refer to various CBT workbooks to devise more comprehensive and integrated counseling plans. Studies have suggested that the benefits of exposure to d-cycloserine (DCS) as an adjuvant may enhance the effectiveness of treatment for anxiety disorders. It also suggests that in future studies, some combination of adjuvant drugs could be tried to improve treatment (Guastella et al., 2008).

## 5 Limitations and future research directions

This study also has some limitations. First, this study involved only one case, and although the treatment effect is supported by evidence, empirical studies from large populations are needed. Second: the measurement instrument in this study is the self-assessment scale, which can be used to measure the treatment effect, but it is subjective. Although changes in mood have been confirmed by interviewers, future research should add more objective measures. Future studies should attempt to examine the therapeutic effects of this counseling regimen in larger Randomized controlled trial across cultures. It is also important to note that, due to the complexity of the interviewer profile, researchers can explore combining CBCFF with additional techniques and methods. By combining the strengths of multiple approaches, it is anticipated that more comprehensive and effective counseling solutions can be developed for individuals who are struggling with anxiety. If cognitive neuroscience can be combined to explore the neural mechanisms behind treatment, it will provide more information for future research.

## Data Availability

The raw data supporting the conclusions of this article will be made available by the authors, without undue reservation.
